# Periodontitis in Pregnant Women: A Possible Link to Adverse Pregnancy Outcomes

**DOI:** 10.3390/healthcare11101372

**Published:** 2023-05-10

**Authors:** Andrea Butera, Carolina Maiorani, Annalaura Morandini, Julia Trombini, Manuela Simonini, Chiara Ogliari, Andrea Scribante

**Affiliations:** 1 Unit of Dental Hygiene, Section of Dentistry, Department of Clinical, Surgical, Diagnostic and Pediatric Sciences, University of Pavia, 27100 Pavia, Italy; 2 Member Association: “Mamme & Igieniste”, 24125 Bergamo, Italy; 3 Policlinico San Matteo, 27100 Pavia, Italy; 4 Unit of Orthodontics and Pediatric Dentistry, Section of Dentistry, Department of Clinical, Surgical, Diagnostic and Pediatric Sciences, University of Pavia, 27100 Pavia, Italy

**Keywords:** periodontitis, pregnancy, oral health, adverse pregnancy outcomes

## Abstract

Background: Periodontitis develops in 11% of pregnant women, and it is independently linked to severe complications during pregnancy such as preterm birth, low birth weight, and gestational diabetes. Methods: A literature search (Pubmed/MEDLINE, and Scopus) from 2003 to 2023 was conducted to analyze studies focused on periodontitis and adverse pregnancy outcomes. Results: 16 articles have been included. Most of the studies showed adverse outcomes, like preterm birth and the low weight of the unborn child, are among the most frequent consequences (respectively 62.5% and 68.7% of articles); pre-eclampsia is also linked to this condition (12.5% of articles); and perinatal mortality (12.5% of articles). Conclusions: Periodontal disease appears to be associated with adverse events in pregnancy due to the transport of biofilm bacteria into the bloodstream and into placental tissue; what would cause adverse events is the body’s immune response to infection.

## 1. Introduction

Periodontal disease is a chronic and irreversible pathology of the supporting tissues of the tooth that affects between 20% and 50% of the world population; the distribution in adults differs significantly in low (28.7%), lower-middle (10%), upper-middle (42.5%), and high-income countries (43.7%) [1,2].

Periodontal disease is one of the risk factors for other systemic diseases, such as pneumonia, diabetes mellitus, arteriosclerosis, and coronary heart disease [3]. Numerous studies show that periodontitis sufferers have higher circulating neutrophil granulocyte values and higher systemic inflammatory parameters (such as reactive protein C) compared to healthy people. In particular, the latter parameter is an excellent predictor for the development of ischemic diseases, atherosclerosis, and the imperfect metabolic control of diabetes [4,5]. In addition, it seems to be one of the risk factors for complications during pregnancy [6].

About 14.2 to 54.8% of pregnant women suffer from periodontal disease, and 11% develop periodontitis, which can cause the destruction of periodontal tissue and the distribution of bacteria and other inflammatory mediators [7].

Periodontitis is independently linked to severe complications during pregnancy, such as preterm birth, low birth weight, and gestational diabetes. [7,8]: the reason could be linked to the translocation of pathogenic bacteria to the fetus-placenta unit or the effect of inflammatory mediators such as interleukin-1 (IL-1), IL-6, IL-8, tumor necrosis factor alpha (TNF alpha), or prostaglandin E2 (PGE2) on the fetus-placenta unit [9,10].

Bacteremia is the transient or continuous presence of viable bacteria in the bloodstream. In people with periodontal disease, subgingival microflora is contracted with the damaged inner epithelium of periodontal pockets, which allows bacteria to enter the bloodstream [11]. This would seem to be the mechanism for the association between periodontal disease and adverse events during pregnancy [12].

Two experimental hypotheses support the correlation between periodontitis and pregnancy-related negative events. The first is based on the possibility that women with periodontitis are subject to frequent bacteremia. Bacteria activate a cascade of inflammatory processes at the level of the placenta and the fetus, with the risk of pre-term delivery and/or birth of underweight children [13]. The second hypothesis is based on the fact that periodontitis can cause a generalized increase in cytokines, substances with pro-inflammatory activity that cause alterations to the placenta and the fetus [14]. The periodontal bacteria (in particular *Porphyromonas Gingivalis*) can in fact enter the bloodstream, reach the placenta, and generate toxins inside the amniotic fluid that result in inflammation, potentially causing premature birth [15]. In addition to premature birth, other related adverse events include reduced body weight gain of the unborn child and the development of premature uterine contractions, with the risk of pre-term delivery and/or the birth of underweight children [16].

The onset of periodontal disease appears to be linked to a change in the composition of sub-bacterial gingival flora with an increase in the relative amount of pathogenic periodontal anaerobic bacteria associated with increased circulating levels of estrogen and progesterone and these would be a factor promoting the growth of pathogenic periodontal anaerobes; alteration of the local immune response with increased susceptibility to gum inflammation and depression of the chemotactic and phagocytic response of neutrophil granulocytes and other cellular-immune functions mediated, which contributes to the stimulation of the production of prostaglandins induced by varicose veins; to the pro-inflammatory effects on the gums mainly on vascular proliferation, on the production of collagen, on epithelial keratinization and the fluid content of the connective tissue [17,18,19].

On the basis of these considerations, a review has been carried out to investigate the correlation between periodontal disease and adverse events in pregnancy, according to the most recent literature.

## 2. Materials and Methods

### 2.1. Focused Question

The present literature review aims to investigate the correlation between periodontal disease and adverse events in pregnancy, according to the most recent literature [20].

### 2.2. Eligibility Criteria

Studies in accordance with the following inclusion criteria:

Type of studies: case-control, cross-sectional, cohort studies, and clinical trials published in English.

Type of participants: pregnant women with periodontal disease.

Type of interventions: case-control, cross-sectional, cohort studies, clinical trials, and reviews that have evaluated the association between periodontal disease and adverse events in pregnancy.

Outcome type: adverse events in pregnancy.

In the second phase, were included studies that met all the inclusion criteria, that is to say, the analysis of the selected studies according to the exclusion criteria: (I) studies where the authors had not reported outcome; (II) in vitro or animal clinical studies; (III) studies carried out without the approval of the Ethics Committee; (IV) reviews and metanalysis.

### 2.3. Search Strategy

The review is based on the research of studies identified through bibliographic research in electronic databases and by examining the bibliographies of articles on Pubmed/MEDLINE, and Scopus. Initially, all study abstracts were taken into consideration.

### 2.4. Research

We performed the search using the following keywords: “pregnancy” AND “periodontal disease”; “pregnancy” AND “periodontitis”; “periodontitis” AND “adverse outcomes” IN “pregnancy”; “periodontitis” AND “low-birth weight” IN “pregnancy”; “periodontitis” AND “pre-term birth” IN “pregnancy”; “periodontitis” AND “pre-eclampsia” IN “pregnancy”; “alterations” IN “oral health” AND “adverse outcomes” IN “pregnancy”. The search has had a similar temporal range from 2003 to 2023.

### 2.5. Screening and Selection of Articles

**Figure 1:**
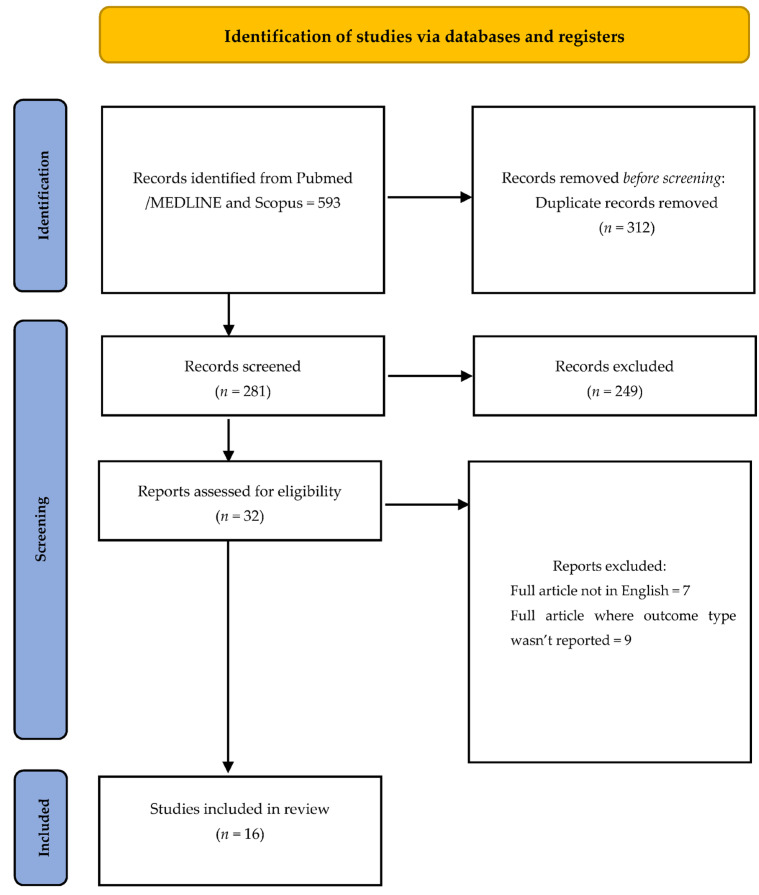
Flow chart of included studies: from 593 articles, duplicates were eliminated, and 281 articles remained; from them, 16 articles were analyzed.

The search produced 593 titles matching the search keywords. The following flowchart shows the selection criteria used to select the final 16 articles that were used for the review analysis. Figure 1. The results were filtered for relevance to the association between periodontitis and adverse outcomes in pregnancy. Articles were analyzed and grouped to assess the possible association between periodontitis and adverse outcomes in pregnancy.

All articles that did not meet the eligibility criteria were rejected.

In the first phase, all the abstracts have been included, and successively, all the duplicate articles from the searches carried out by three auditors independently are excluded.

At a later stage, abstracts were analyzed by the same authors for consistency with the eligibility criteria; therefore, at this stage, all articles that were not case-control, cross-sectional, cohort studies, clinical trials, or any articles that did aim to evaluate periodontal disease and its effects on adverse events in pregnancy are excluded. Ultimately, the remaining articles were read in full. A further skimming was therefore carried out: some selected articles were not available in English, and some (not having mentioned the objective in the abstract) were excluded because they were not focused on the association between periodontal disease and adverse events in pregnancy. The last phase, that of reading and ulterior exclusion, has been carried out by two other reviewers not involved in the first two phases.

### 2.6. Risk of Bias and Results

From the analysis of the articles, a reviewer was concerned to highlight the results obtained from reading the selected articles. Articles have been included in a table on the basis of the adverse event found and its association with periodontal disease or not. Then a final reviewer evaluated the risk of bias of each study involved; the quality of the studies has been assessed on the basis of the information provided [21].

## 3. Results

In pregnant women with periodontal disease, some adverse events were found. Among these, preterm birth and the low weight of the unborn child are among the most frequent consequences (respectively 50% and 50% of articles); pre-eclampsia is also linked to this condition (12.5% of articles); and perinatal mortality (12.5% of articles) [22,23,24,25,26,27,28,29,30,31,32,33,34,35,36,37] Table 1.

### Risk of Bias

Randomization, allocation concealment, blinding, outcome data, and outcome recording were evaluated; a color was assigned according to the type of risk. The green symbol was assigned where the information was complete according to the variable considered (low risk of bias); the yellow symbol was assigned where the information was missing or not clear (moderate risk of bias) [21].

Table 2 shows the risk of bias in the main articles examined; this review presents a moderate risk of bias.

## 4. Discussion

Several studies have shown the onset of periodontal problems during the second and third trimesters of pregnancy due to the change and increase in sex hormones and blood flow.

High levels of estrogen can cause gingival hypersensitivity to local factors, including bacterial plaque. This can lead to an increase in gingival volume, often associated with bleeding, or even the onset of periodontal disease, which usually regresses at the end of pregnancy; this is due to the growth of anaerobic bacteria, associated with increased circulating levels of estrogen but also progesterone [38,39].

Moreover, during gestation, the alteration of the vascular permeability of the gums can facilitate the transport of biofilm bacteria into the bloodstream until they reach the placental tissue [38].

In this tissue, the slow venous circulation and the invasive ability of microorganisms promote possible penetration into the fetus and into the amniotic fluid. Here, an immune response is triggered that could lead to the release of pro-inflammatory cytokines. If the body is able to fight the infection, there will be no consequence; otherwise, membrane rupture and premature birth may occur. These inflammatory compounds can negatively regulate the expression of genes essential for the growth of the fetus, causing a low birth weight, and generating structural damage to the placental circulation that increases the blood pressure of the mother [13,14,15,16].

The results of the articles analyzed in this review are in line with what is already present in the literature [40,41,42,43,44,45].

Periodontal disease in pregnant women would appear to develop unexpected and adverse results, such as preterm birth [25,28,29,30,32,35,36,37], low birth weight [24,25,26,30,32,33,36,37], and pre-eclampsia [23]. The objective of the review was to research and highlight possible adverse events in pregnancy in women with periodontal disease. Preterm birth and low birth weight appear to be the most common events, at least according to this analysis. Preterm birth was found in 50% of the studies analysed, a result similar to that obtained by Manrique-Corredor et al. in a systematic and meta-analytical review, the authors evaluated 31 studies, and 60% of them saw an association between periodontal disease and preterm birth; Chambrone et al. also showed a positive association with preterm birth and low birth weight in 81.8% of the studies involved in the review [46,47].

The analyzed studies support these results: 50% of the studies showed an association between periodontal disease and preterm birth, and 50% had an association with low birth weight.

Periodontal diseases are associated with an increased risk of premature and/or underweight births [48]. Today, it is known that some acute inflammatory processes in the mother, even if localized far from the genitourinary tract, can play a secondary role in the appearance of pathological alterations in pregnancy [49]. Other studies, however, have not shown a clear association between periodontal disease and any adverse events [22,27,31,34].

However, although these events have often been associated, there is no clear evidence or correlation between periodontal disease and pregnancy. A predisposition can be affirmed due to the migration of bacteria present at the periodontal pockets through the bloodstream to the placenta and the fetus, which could cause muscular contractions of the uterus, cervical dilation, and premature rupture of the amniotic sac [50]. Numerous clinical studies would be needed to establish a positive association between periodontal disease and adverse events during pregnancy.

So, based on these considerations, pregnant women and women planning a pregnancy should be aware that there may be a link between their periodontal condition, general health, and possible complications of pregnancy. For this reason, periodontal treatment may be necessary, which in this case should be performed before conception; however, it could also be performed during pregnancy [51]. In addition, as negative pregnancy outcomes and periodontitis have in common some important risk factors (e.g., smoking), pregnant women should be aware of the importance of healthy habits and lifestyles [52]. It has been shown that periodontal treatment carried out in pregnancy is safe and helps to reduce the level of gingival inflammation, allowing oral health to be maintained in this delicate period. In addition, current scientific knowledge suggests that periodontal therapy before conception may reduce the risk of the related adverse effects mentioned above [8,51].

Unfortunately, very often treatments are carried out late; the ideal would be to start before pregnancy to obtain a positive outcome on the clinical result of gestation. In fact, non-surgical periodontal treatment in pregnant women would seem not to achieve an improvement of the unfavorable, probably also because the available studies are very heterogeneous. The result is mixed opinions [40,53,54,55,56,57].

However, action should be taken against dysbiosis caused by periodontal disease, perhaps by supplementing the use of probiotics in pregnant women, which seem to have positive effects on gum bleeding indices, together with the correct oral hygiene methods for the removal of bacterial biofilm [38,58,59].

The studies involved in this review present some limitations. Although most of them have validated the association between periodontal disease and some adverse events in pregnancy, this statement cannot be generalized. The results of the individual studies are influenced by some variables such as ethnicity, socio-economic status, age, the period of pregnancy, and systemic pathologies related to the mother. It would be useful to standardize the sample taken from the studies, trying to eliminate any variable that could influence the results.

Therefore, as the studies evaluated different variables, a direct comparison is not possible. Additionally, it has been noticed that there is a lack of homogeneity among the measurements of the variables in the studies included in this review. Therefore, future clinical studies with the evaluation of the same variables are needed in order to allow a more complete review, which also requires a meta-analysis.

## 5. Conclusions

Periodontal disease appears to be associated with adverse events in pregnancy due to the transport of biofilm bacteria into the bloodstream and into placental tissue; what would cause adverse events is the body’s immune response to infection.

Preterm birth and low birth weight seem to be associated with periodontal disease in pregnant women. Although numerous studies are needed to define a significant positive association.

In light of this link, it is important to assess the oral health of pregnant women in order to intercept any risky situations.

## Figures and Tables

**Table 1:** Articles examined.

Authors	Type of Study	(Problem-Population)	Intervent/ Control	Outcomes	Association
Santa Cruz et al., 2013 [26]	Cohort study	Problem: the association between periodontal disease, and adverse pregnancy outcomes. Population: 170 women were included (mean age 31.9, rangin 20–40) between the 8th to 26th wk of pregnancy	Clinical parameters: PI, BoP, PPD, Gingival recession, Microbiological samples. Demographic and medical data: gestational age, race, maternal weight before pregnancy, maternal height, previous deliveries, previous PTB or LBW, maternal diseases, metabolic or genetic alterations, socio-economic, and educational status	The periodontal condition was not associated with adverse pregnancy: preterm birth, low-weight- birth, preterm and low-birth weight and preterm or low-birth weight.	No
Boggess et al., 2006 [27]	Cohort study	Problem: the association between periodontal disease and delivery of a small-for-gestational-age infant (less than the 10th percentile for gestational age) Population: 1017 women were included	Clinical parameters: PPD, CAL, BoP. Demographic and medical data: gestational age, maternal weight, previous deliveries less than 37th, deliveries less than 37th, pre-eclampsia, tobacco, alcohol, and drug consumption.	The periodontal condition was associated with delivery of a small-for-gestational-age infant. The small-for-gestational-age rate was higher among women with moderate or severe periodontal disease, compared with those with health or mild disease (13.8% versus 3.2% versus 6.5%, *p* < 0.001). Moderate or severe periodontal disease was associated with a small-for-gestational-age infant, a risk ratio of 2.3 (1.1 to 4.7).	Yes
Saddki et al., 2008 [28]	Cohort study	Problem: the association between periodontal disease, and low birth weight Population: 472 women were included (ranges from 14 to 46 years old) in the second trimester of pregnancy	Clinical parameters: PPD, CAL, BoP. Demographic and medical data: haemoglobin level, rate of weight gain, history of pre-term birth, history of abortion, history of low-birth weight, socio-economic, and educational status	After adjustment for potential confounders using multiple logistic regression analysis, significant association was found between maternal periodontitis and LBW (OR = 3.84; 95% CI: 1.34–11.05).	Yes
Kumar et al., 2013 [29]	Cohort study	Problem: the association between periodontal disease, and adverse pregnancy outcomes. Population: 340 women were included (ranges from 18 to 35 years old) at 14–20 weeks of pregnancy	Clinical parameters: BoP, PPD, CAL, Gingival recession. Demographic and medical data: gestational age, socio-economic and educational status, pre-eclampsia, IUGR abruption placenta, type of labor, ode of delivery, neonatal outcome, and birthweight	The study shows a significant association between periodontitis (but not with gingivitis) and adverse pregnancy outcomes. Maternal periodontitis is associated with an increased risk of pre-eclampsia, intrauterine growth restriction, preterm delivery and low birthweight infants with odds ratios (95% confidence interval) of 7.48 (2.72–22.42), 3.35 (1.20–9.55), 2.72 (1.30–5.68) and 3.03 (1.53–5.97), respectively.	Yes
Marin et al., 2005 [30]	Cross-sectional study	Problem: the association between periodontal disease and low birth weight Population: 152 women were included (ranges from 14 to 39 years old)	Clinical parameters: PI, BoP, PPD, CAL. Demographic and medical data: gestational age, educational status, maternal height, previous live births, previous aborts, previous preterm low birth weight, gestational age, maternal gain in weight, infant birth weight, tobacco, alcohol, and drug consumption.	Periodontal disease in pregnant women is statistically associated with a reduction in the infant birth weight.	Yes
Srinivas et al., 2009 [31]	Cohort study	Problem: the association between periodontal disease and adverse pregnancy outcomes (preterm birth, preeclampsia, fetal growth restriction, or perinatal death) Population: 152 women were included	Clinical parameters: PPD, CAL. Demographic and medical data: gestational age, maternal height, previous live births, previous abortions, previous preterm deliveries, pre-eclampsia	There was no association between PD and the composite outcome (adjusted odds ratio [AOR], 0.81; 95% confidence interval [CI], 0.58–1.15; *p* = 0.24), preeclampsia (AOR, 0.71; 95% CI, 0.37–1.36; *p* = 0.30), or preterm birth (AOR, 0.77; 95% CI, 0.49–1.21; *p* = 0.25) after adjusting for relevant confounders.	No
Agueda et al., 2008 [32]	Cohort study	Problem: the association between periodontal disease and preterm birth, low birth weight, and preterm low birth weight Population: 1296 women were included	Clinical parameters: PI, BoP, PPD, CAL. Demographic and medical data: socio-economic and educational status, residence, ethnicity, body mass index, previous preterm delivery, previous low birth weight, previous miscarriage, pregnancy complications, gestational diabetes, caesarian delivery, antibiotic intake, systemic diseases, and tobacco consumption	The factors involved in many cases of adverse pregnancy outcomes have still not been identified, although systemic infections may play a role. This study found a modest association between periodontitis and PB. Further research is required to establish whether periodontitis is a risk factor for PB and/or LBW.	Yes
Offenbacher et al., 2006 [33]	Cohort study	Problem: the association between periodontal disease and preterm birth, low birth weight, and preterm low birth weight Population: 1020 women were included before 26 weeks of pregnancy	Clinical parameters: PI, BoP, PPD, CAL. Demographic and medical data: maternal age, maternal weight, previous preterm delivery, medical insurance, tobacco, alcohol, and drug consumption.	Antepartum moderate-severe periodontal disease was associated with an increased incidence of spontaneous preterm births (15.2% versus 24.9%, adjusted RR 2.0, 95% CI 1.2–3.2). Similarly, the unadjusted rate of very preterm delivery was 6.4% among women with periodontal disease progression, significantly higher than the 1.8% rate among women without disease progression (adjusted RR 2.4, 95% CI 1.1–5.2).	Yes
Rakoto-Alson et al., 2010 [34]	Cohort study	Problem: the association between periodontal disease and preterm birth, and low birth weight Population: 204 women were included (25.6 years old) at 20–34 weeks of pregnancy	Clinical parameters: PI, PBI, PPD. Demographic and medical data: socio-economic and educational status, gestational age, birth weight, type of delivery, and previous pregnancy.	Periodontitis (at least three sites from different teeth with clinical AL > or = 4 mm) was significantly associated with PB (*p* < 0.001), LBW (*p* < 0.001), and PLBW (*p* < 0.01). The rates of periodontitis were considerably higher in the PB (78.6%), LBW (77.3%), and PLBW (77.8%) groups than in the full-term (8.6%), normal weight (16.5%), and normal birth (2.7%) groups.	Yes
Moore et al., 2004 [35]	Cohort study	Problem: the association between periodontal disease and preterm birth, low birth weight, and late miscarriage Population: 3738 women were included (29.5 years old) at 12 weeks of pregnancy	Clinical parameters: PI, BS, PPD, CAL. Demographic and medical data: socio-economic status, ethnicity, previous preterm delivery, previous miscarriage, medications in 1st trimester, antibiotics in 1st trimester, urinary tract infection in 1st trimester, alcohol, and tobacco consumption.	Regression analysis indicated that there were no significant relationships between the severity of periodontal disease and either preterm birth (PTB) or low birth weight (LBW). In contrast, there did appear to be a correlation between poorer periodontal health and those that experienced a late miscarriage.	No
Ercan et al., 2013 [36]	Cohort study	Problem: the association between periodontal disease and preterm birth, low birth weight, and late miscarriage Population: 50 women undergoing amniocentesis were included	Clinical parameters: PI, BoP, PPD, CAL, GI, microbiological samples. Demographic and medical data: marital and educational status, gestational age, and birth weight	The transmission of some periodontal pathogens from the oral cavity of the mother may cause adverse pregnancy outcomes. The results contribute to an understanding of the association between periodontal disease and PTLBW, but further studies are required to better clarify the possible relationship.	Yes
Moreu et al., 2005 [37]	Cohort study	Problem: the association between periodontal disease and preterm birth, and low birth weight Population: 96 women were included (ranges from 18 to 40 years old)	Clinical parameters: PI, GI, PPD. Demographic and medical data: unknown	A relationship was observed between low-weight birth and probing depth measurements, especially the percentage of sites of >3 mm depth, which was statistically significant (*p* = 0.0038) even when gestational age was controlled for.	Yes
Mobeen et al., 2008 [38]	Cohort study	Problem: the association between periodontal disease, and birth outcomes. Population: 1037 women were included	Clinical parameters: PI, GI, PPD, CAL. Demographic and medical data: ethnicity, educational status, number of pregnancies, previous miscarriage/abortion, previous stillbirth	As various measures of the severity of the periodontal disease increased, both stillbirth and neonatal death increased, accompanied by a non-significant increase in early preterm birth. It is unknown if treatment of periodontal disease either before or during pregnancy would improve these adverse pregnancy outcomes.	No
Lopez et al., 2008 [39]	Cohort study	Problem: the association between periodontal disease, and adverse pregnancy outcomes. Population: 1404 women were included	Clinical parameters: PPD, CAL, BoP. Demographic and medical data: systemic disease, onset of prenatal care, previous PB, complications of pregnancy, and type of delivery.	This study found a modest association between periodontitis and PB.	Yes
Vogt et al., 2010 [40]	Cohort study	Problem: the association between periodontal disease, and adverse pregnancy outcomes. Population: 327 women were included (ranges from 18 to 42 years old) before 32 weeks of pregnant	Clinical parameters: PI, GR, PPD, CAL, BoP. Demographic and medical data: socio-demographic variables (age, parity, race/color, years of schooling, marital status, body mass index-BMI-estimated with the pregnancy weight, and any systemic diseases); habit variables (smoking and alcohol consumption); and gestacional variables (number of prenatal visits, bacterial vaginosis, vaginal delivery, and the newborn Apgar scores at the first and fifth minute of life).	PD was associated with a higher risk of PTB (RR_adj._ 3.47 95%CI 1.62–7.43), LBW (RR_adj._ 2.93 95%CI 1.36–6.34) and PROM (RR_adj._ 2.48 95%CI 1.35–4.56), but not with SGA neonates (RR 2.38 95%CI 0.93–6.10).	Yes
Turton et al., 2017 [41]	Cohort study	Problem: the association between periodontal disease, and adverse pregnancy outcomes. Population: 443 women were included (ranges from 18 to 42 years old)	Clinical parameters: PI, GI, CAL. Demographic and medical data: age, race, educational level, stage of pregnancy, and medical history.	Significant associations were found between pregnancy outcomes and maternal periodontal index scores (low birth weight and preterm delivery).	Yes

**Table 2:** Risk of bias in the articles examined; green symbol indicates a low risk of error, while yellow symbol indicates moderate risk of bias.

Articles	Adequate Sequence Generated	Allocation Concealment	Blinding	Incomplete Outcome Data	Registration Outcome Data
Santa Cruz et al., 2013 [26]	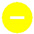	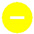	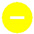	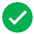	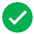
Boggess et al., 2006 [27]	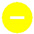	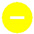	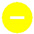	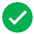	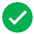
Saddki et al., 2008 [28]	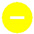	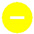	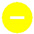	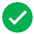	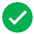
Kumar et al., 2013 [29]	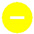	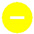	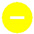	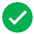	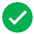
Marin et al., 2005 [30]	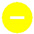	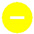	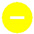	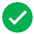	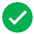
Srinivas et al., 2009 [31]	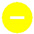	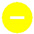	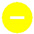	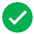	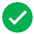
Agueda et al., 2008 [32]	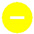	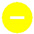	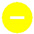	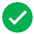	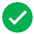
Offenbacher et al., 2006 [33]	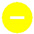	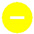	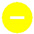	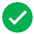	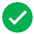
Rakoto-Alson et al., 2010 [34]	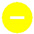	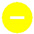	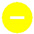	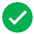	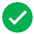
Moore et al., 2004 [35]	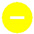	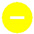	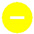	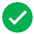	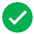
Ercan et al., 2013 [36]	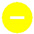	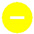	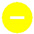	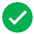	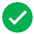
Moreu et al., 2005 [37]	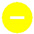	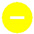	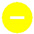	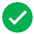	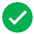
Mobeen et al., 2008 [38]	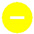	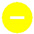	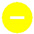	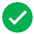	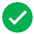
Lopez et al., 2008 [39]	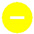	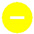	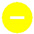	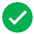	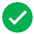
Vogt et al., 2010 [40]	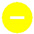	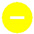	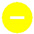	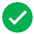	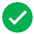
Turton et al., 2017 [41]	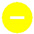	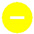	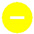	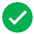	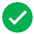

## Data Availability

The data presented in this study are available on request from the corresponding author.
